# Long-term osteoporosis treatment: myth or reality?

**DOI:** 10.1186/ar3709

**Published:** 2012-03-08

**Authors:** Andrea Giusti

**Affiliations:** 1 Bone Clinic, Department of Gerontology and Musculoskeletal Sciences, Galliera Hospital, Genova, Italy

## 

Several pharmacological agents [bisphosphonates (BPs), SERMs, teriparatide, PTH 1-84, strontium ranelate, denosumab] have been approved worldwide for the prevention of fragility fractures in patients at risk. In pivotal randomized-controlled trials (RCTs), these drugs demonstrated to reduce the incidence of new vertebral fractures, and, in some cases, of new non-vertebral and hip fractures, in women and men with primary and glucocorticoid-induced osteoporosis, with a good profile of safety and tolerability. Most of the studies, however, were designed to assess the efficacy and safety of these drugs for 3 to 5 years, and only few of them extended over 5 years (BPs).

Although RCTs extension studies were carried out in smaller samples compared to the original baseline populations enrolled, their results support the sustained beneficial effects on skeletal metabolism of alendronate (10 years), zoledronate (6 years) and risedronate (7 years) on the long term. Due to their pharmacological properties, BPs have also demonstrated to prolong their efficacy after discontinuation in specific subgroups of patients.

In recent years, some concerns have been raised about long-term safety of BPs, due to "unexpected" rare adverse events (AEs) potentially associated with their use (atypical fractures, ONJ and esophageal cancer). Indeed, a cause-effect relationship has not been yet demonstrated. However, given the dramatic implications of these rare AEs, a drug-free holiday should be considered in patients treated for more than 5 years with BPs, after an accurate evaluation of risks and benefits.

Regarding the recently approved anti-osteoporotic agents less information are available. Denosumab have been shown to produce a sustained increase of bone mineral density over 8 years of treatment, and to reduce the risk of new fragility fracture up to 5 years of continuous treatment. Similar results have been described also for strontium ranelate.

In this context, studies are warranted to clarify when and for how long a drug-free holiday should be considered in patients receiving BPs. Moreover further trials are needed to clarify the long-term safety and beneficial effects of the newly approved agents (denosumab, strontium ranelate).

